# Evidence from 617 laboratories in 47 countries for SLMTA-driven improvement in quality management systems

**DOI:** 10.4102/ajlm.v3i2.262

**Published:** 2014-11-03

**Authors:** Katy Yao, Elizabeth T. Luman, SLMTA Collaborating Authors

**Affiliations:** 1 International Laboratory Branch, Division of Global HIV/AIDS, US Centers for Disease Control and Prevention (CDC), Atlanta, Georgia; 2 See Appendix: Author information

## Abstract

**Background:**

The Strengthening Laboratory Management Toward Accreditation (SLMTA) programme is a large-scale effort to improve the quality of laboratories in resource-limited countries.

**Objectives:**

This study sought to evaluate the first four years (2010–2013) of SLMTA implementation.

**Methods:**

Country-level data were submitted by SLMTA programme leads and compiled globally. Performance was measured before (baseline) and after (exit) SLMTA implementation using an audit checklist which results in a percentage score and a rating of zero to five stars. Some laboratories continued to monitor performance in post-exit surveillance audits. We evaluated score improvements using two-tailed *t*-tests for equal variances and estimated the number of tests performed by SLMTA laboratories based on star level.

**Results:**

SLMTA was implemented in 617 laboratories in 47 countries in Africa, the Caribbean, Latin America and Southeast Asia. At the baseline audit, the laboratories scored an average of 39% on the checklist and 84% of them were rated below one star. As of December 2013, 302 laboratories had completed the SLMTA programme; mean checklist scores increased from 39% at baseline to 64% at exit (*p* < 0.001) over an average 16-month programme duration. Ninety-two laboratories conducted a surveillance audit at a median of 11 months after their exit audit; 62% further increased their performance. Six SLMTA laboratories have achieved accreditation status. In total, the 617 SLMTA laboratories conduct an estimated 111 million tests annually. Only 16% of these tests were conducted by laboratories with at least one star at baseline, which increased to 68% of tests after SLMTA training. Thus, approximately 23 million tests are conducted annually by laboratories previously at zero stars that now have one to five stars; this number is projected to increase to 58 million when currently-enrolled laboratories complete the programme.

**Conclusion:**

SLMTA has transformed the laboratory landscape in resource-limited countries worldwide and has the potential to make a substantial and sustainable impact on the quality of laboratory testing and patient care.

## Introduction

Substantial resources have been invested in management training and development to help scale up and improve the quality of health services in order to reach the United Nation’s Millennium Development Goals.^1^ However, evidence is scarce in terms of the impact that has actually been achieved. An international meeting convened by the World Health Organization (WHO) in 2005 on strengthening leadership and management in low-income countries concluded that programme evaluation often focuses on the number of trainees and pre-/post-knowledge-based tests; evaluation of programme impact on managers’ daily work and job outcomes is more the exception than the norm.^2^

Strengthening Laboratory Management Toward Accreditation (SLMTA) is a large-scale effort aimed at improving the quality of laboratory services and patient care in resource-limited settings by developing competent laboratory managers. SLMTA provides an innovative training curriculum on implementing practical Quality Management Systems (QMS) using existing resources with built-in accountability and evaluation that focuses on result-oriented outcome measures.^3^ Launched in 2009 in Kigali, Rwanda, this programme seeks to engage laboratories in continuous quality improvement and to accelerate their preparations toward accreditation to international standards.^4,5^

In this article, we present evidence from the first four years of SLMTA programme implementation (2010–2013). We report data on changes in levels of laboratory compliance with International Organization for Standardization (ISO) 15189 requirements using the WHO Regional Office for Africa’s (WHO AFRO) accreditation preparedness checklist. The findings from this unprecedented study shed light on the widespread success of an innovative programme that has empowered laboratory teams throughout the developing world to strive for continuous quality improvement and work toward accreditation, despite limited resources.

## Research methods and design

### SLMTA implementation and evaluation

The methodology of the SLMTA programme has been described previously.^3,5^ Briefly, SLMTA is a competency-based programme that uses a series of short courses and work-based learning projects to effect rapid and measurable laboratory improvement for better patient care through enhanced management skills and implementation of practical quality management systems. The SLMTA training programme is based on a series of three workshops. After each workshop, participants implement improvement projects supported by regular supervisory visits or on-site mentoring. Laboratories are evaluated using WHO AFRO’s Stepwise Laboratory Quality Improvement Process Towards Accreditation (SLIPTA) checklist,^6^ which includes 111 items divided into 12 sections that represent the 12 Quality System Essentials (QSEs).^7^ In order to assess progress made by the laboratories, audits are conducted using the SLIPTA checklist at the beginning (baseline) and at the end (exit) of the SLMTA programme. Many countries also conduct intermediate audits to help guide programme implementation, as well as surveillance audits after exiting the programme so as to monitor continued improvement and assess sustainability. After an audit, laboratories receive a score which determines their star rating – from zero to five, with < 55% corresponding to zero stars, 55% – 64% one star, 65% – 74% two stars, 75% – 84% three stars, 85% – 94% four stars and ≥ 95% five stars. When a laboratory achieves a five-star rating, it may be encouraged to seek accreditation.

### Data analysis

Programme data up to December 2013 were collected from all countries implementing SLMTA. Variables included year of implementation, number and types of laboratories in each cohort (i.e., enrolment in the same training round), number of people trained, audit scores and approximate number of tests conducted by each laboratory. Data were collated and analysed in Microsoft^®^ Excel 2013. Descriptive statistics (percentages, medians, ranges) were calculated. Statistical significance of improvements were assessed using 2-tailed *t*-tests for equal variances (*f*-tests showed equal variances at *p* > 0.1); comparison of improvements across laboratory types was assessed using one-way analysis of variance (ANOVA).

Given the wide variations in the time between the baseline audit and the first SLMTA workshop, programme length was defined as the time from the first SLMTA workshop to the exit audit. In some countries, results for large national reference laboratories were reported by department rather than for the laboratory as a whole. For consistency, we aggregated the department scores into a single score per laboratory, using median values across laboratory departments. We estimated the number of laboratory tests conducted in SLMTA laboratories based on country reports for 2012; missing data were imputed using averages by laboratory type.

## Results

### Programme spread

**FIGURE 1:**
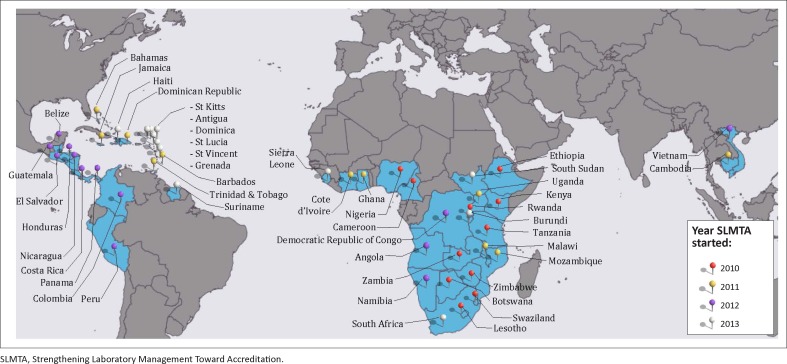
Global reach of SLMTA by year of implementation, 2010−2013 (*n* = 47 countries).

Since its introduction in 2009, SLMTA has been implemented in 47 countries worldwide, including 23 countries in Africa, 12 in the Caribbean Region, 10 in Central and South America, and two in Southeast Asia (Figure 1, Table 1).

**TABLE 1:** Strengthening Laboratory Management Toward Accreditation: Training and Results, 2010-2013.

Country	Number of Laboratories Participating*	Cohort	Year Implemented	# of Laboratories in Cohort	Type of Laboratory						# of People Trained in Cohort	Baseline Audit Score Median (%)	Baseline Stars						# of Laboratories Completed Exit Audit	Exit Audit Score Median (%)	Difference in Medians of Audit Scores (%)**	Exit Stars					
					National	Regional or Provincial	District or Primary	NGO, Faith-Based, or Private	Military	Blood Bank			0	1	2	3	4	5				0	1	2	3	4	5
Angola	5	-	-	-	-	-	-	-	-	-	-	-	-	-	-	-	-	-	-	-	-	-	-	-	-	-	-
	-	1	2012	1	1	0	0	0	0	0	15	24	1	0	0	0	0	0	-	-	-	-	-	-	-	-	-
	-	2	2013	4	0	0	4	0	0	0	24	16	4	0	0	0	0	0	-	-	-	-	-	-	-	-	-
Botswana	28	-	-	-	-	-	-	-	-	-	-	-	-	-	-	-	-	-	-	-	-	-	-	-	-	-	-
	-	1	2010	8	5	0	3	0	0	0	24	54	4	3	1	0	0	0	7	71	17	2	0	4	1	0	0
	-	2	2011	10	2	0	8	0	0	0	20	54	6	1	2	1	0	0	-	-	-	-	-	-	-	-	-
	-	3	2012	12	1	0	6	1	4	0	24	38	11	0	1	0	0	0	-	-	-	-	-	-	-	-	-
Burundi	6	-	-	-	-	-	-	-	-	-	-	-	-	-	-	-	-	-	-	-	-	-	-	-	-	-	-
	-	1	2012	6	2	0	4	0	0	0	24	24	6	0	0	0	0	0	6	64	40	2	1	3	0	0	0
Cambodia	7	-	-	-	-	-	-	-	-	-	-	-	-	-	-	-	-	-	-	-	-	-	-	-	-	-	-
	-	1	2011	7	1	4	2	0	0	0	18	26	7	0	0	0	0	0	7	65	39	0	3	2	2	0	0
Cameroon	14	-	-	-	-	-	-	-	-	-	-	-	-	-	-	-	-	-	-	-	-	-	-	-	-	-	-
	-	1	2010	5	0	4	0	1	0	0	67	23	5	0	0	0	0	0	5	68	45	0	2	2	1	0	0
	-	2	2013	7	5	1	0	1	0	0	110	37	7	0	0	0	0	0	2	61	24	0	2	0	0	0	0
	-	3	2013	2	2	0	0	0	0	0	26	55	1	1	0	0	0	0	-	-	-	-	-	-	-	-	-
Caribbean Region^1^	12	-	-	-	-	-	-	-	-	-	-	-	-	-	-	-	-	-	-	-	-	-	-	-	-	-	-
	-	1	2011	5	5	0	0	0	0	0	24	38	5	0	0	0	0	0	5	60	22	2	2	0	1	0	0
	-	2	2013	7	7	0	0	0	0	0	14	39	7	0	0	0	0	0	-	-	-	-	-	-	-	-	-
Central America Region^2^	8	-	-	-	-	-	-	-	-	-	-	-	-	-	-	-	-	-	-	-	-	-	-	-	-	-	-
	-	1	2012	4	4	0	0	0	0	0	6	55	2	1	1	0	0	0	-	-	-	-	-	-	-	-	-
	-	2	2012	4	4	0	0	0	0	0	6	50	4	0	0	0	0	0	-	-	-	-	-	-	-	-	-
Cote d’Ivoire	21	-	-	-	-	-	-	-	-	-	-	-	-	-	-	-	-	-	-	-	-	-	-	-	-	-	-
	-	1	2012	21	6	5	9	0	0	1	40	39	16	4	0	1	0	0	21	77	38	1	2	5	6	6	1
US-DOD/NMRU^3^	14	-	-	-	-	-	-	-	-	-	-	-	-	-	-	-	-	-	-	-	-	-	-	-	-	-	-
	-	1	2012	14	2	0	0	0	11	1	25	46	9	2	0	1	0	0	-	-	-	-	-	-	-	-	-
Dominican Republic	16	-	-	-	-	-	-	-	-	-	-	-	-	-	-	-	-	-	-	-	-	-	-	-	-	-	-
	-	1	2011	4	3	1	0	0	0	0	25	56	2	1	0	1	0	0	4	70	14	0	0	3	0	1	0
	-	2	2012	4	2	2	0	0	0	0	23	58	1	3	0	0	0	0	4	75	17	0	1	1	1	1	0
	-	3	2012	4	0	4	0	0	0	0	20	45	4	0	0	0	0	0	4	47	2	3	1	0	0	0	0
	-	4	2012	4	0	4	0	0	0	0	14	26	4	0	0	0	0	0	4	65	39	0	1	3	0	0	0
DRC	6	-	-	-	-	-	-	-	-	-	-	-	-	-	-	-	-	-	-	-	-	-	-	-	-	-	-
	-	1	2013	6	3	0	1	1	0	1	26	44	4	2	0	0	0	0	-	-	-	-	-	-	-	-	-
Ethiopia	81	-	-	-	-	-	-	-	-	-	-	-	-	-	-	-	-	-	-	-	-	-	-	-	-	-	-
	-	1	2010	23	9	8	3	0	3	0	60	40	23	0	0	0	0	0	23	59	19	9	8	3	2	1	0
	-	2	2011	21	0	9	11	0	1	0	60	43	20	1	0	0	0	0	21	55	12	11	7	2	1	0	0
	-	3	2013	15	2	5	0	5	2	1	30	30	15	0	0	0	0	0	-	-	-	-	-	-	-	-	-
	-	4	2013	14	0	0	14	0	0	0	28	41	14	0	0	0	0	0	-	-	-	-	-	-	-	-	-
	-	5	2013	8	0	0	8	0	0	0	16	35	8	0	0	0	0	0	-	-	-	-	-	-	-	-	-
Ghana	15	-	-	-	-	-	-	-	-	-	-	-	-	-	-	-	-	-	-	-	-	-	-	-	-	-	-
	-	1	2011	4	2	2	0	0	0	0	20	42	4	0	0	0	0	0	4	67	26	1	1	1	1	0	0
	-	2	2012	5	1	4	0	0	0	0	25	17	5	0	0	0	0	0	-	-	-	-	-	-	-	-	-
	-	3	2013	6	1	5	0	0	0	0	28	13	5	0	0	1	0	0	-	-	-	-	-	-	-	-	-
Haiti	4	-	-	-	-	-	-	-	-	-	-	-	-	-	-	-	-	-	-	-	-	-	-	-	-	-	-
	-	1	2013	4	1	3	0	0	0	0	23	13	3	1	0	0	0	0	-	-	-	-	-	-	-	-	-
Kenya	57	1	2010	12	6	5	1	0	0	0	26	32	12	0	0	0	0	0	12	75	43	0	3	3	4	1	1
	-	2	2011	8	0	5	3	0	0	0	16	36	8	0	0	0	0	0	8	86	50	2	0	0	2	**4**	0
	-	3	2012	7	1	0	4	2	0	0	14	30	6	0	0	1	0	0	-	-	-	-	-	-	-	-	-
	-	4	2012	9	0	0	7	2	0	0	43	36	8	1	0	0	0	0	-	-	-	-	-	-	-	-	-
	-	5	2013	14	0	0	13	0	1	0	39	14	14	0	0	0	0	0	-	-	-	-	-	-	-	-	-
	-	6	2013	7	0	0	0	0	0	7	21	41	7	0	0	0	0	0	-	-	-	-	-	-	-	-	-
Lesotho	20	-	-	-	-	-	-	-	-	-	-	-	-	-	-	-	-	-	-	-	-	-	-	-	-	-	-
	-	1	2010	5	1	0	3	0	1	0	9	39	4	1	0	0	0	0	5	72	33	0	2	0	3	0	0
	-	2	2010	16	1	0	13	2	0	0	16	34	16	0	0	0	0	0	16	59	25	5	5	4	2	0	0
	-	3	2011	19	0	15	3	0	1	0	20	68	3	5	6	4	1	0	19	75	7	1	5	3	6	4	0
Malawi	41	-	-	-	-	-	-	-	-	-	-	-	-	-	-	-	-	-	-	-	-	-	-	-	-	-	-
	-	1	2011	27	2	4	11	8	1	1	50	38	26	0	0	1	0	0	27	48	10	19	5	1	1	1	0
	-	2	2012	19	2	3	6	8	0	0	31	33	16	1	1	0	0	0	-	-	-	-	-	-	-	-	-
Mozambique	19	1	2011	7	0	4	3	0	0	0	25	28	7	0	0	0	0	0	6	58	30	1	3	1	1	0	0
	-	2	2012	12	0	5	5	0	0	2	96	30	12	0	0	0	0	0	-	-	-	-	-	-	-	-	-
Namibia	6	-	-	-	-	-	-	-	-	-	-	-	-	-	-	-	-	-	-	-	-	-	-	-	-	-	-
	-	1	2012	6	0	2	0	2	1	1	8	62	1	4	0	1	0	0	-	-	-	-	-	-	-	-	-
Nigeria	30	-	-	-	-	-	-	-	-	-	-	-	-	-	-	-	-	-	-	-	-	-	-	-	-	-	-
	-	1	2010	24	5	10	3	3	3	0	25	63	7	5	8	3	1	0	24	90	27	0	1	2	3	14	4
	-	2	2013	6	1	0	2	3	0	0	18	42	5	1	0	0	0	0	-	-	-	-	-	-	-	-	-
Rwanda	15	-	-	-	-	-	-	-	-	-	-	-	-	-	-	-	-	-	-	-	-	-	-	-	-	-	-
	-	1	2010	5	1	0	3	0	1	0	21	43	4	0	1	0	0	0	5	73	30	0	1	2	2	0	0
	-	2	2012	5	0	0	5	0	0	0	21	22	5	0	0	0	0	0	5	70	48	0	0	3	2	0	0
	-	3	2013	5	0	0	5	0	0	0	24	32	5	0	0	0	0	0	-	-	-	-	-	-	-	-	-
Sierra Leone	2	-	-	-	-	-	-	-	-	-	-	-	-	-	-	-	-	-	-	-	-	-	-	-	-	-	-
	-	1	2012	1	1	0	0	0	0	0	9	NA	-	-	-	-	-	-	-	-	-	-	-	-	-	-	-
	-	2	2013	2	2	0	0	0	0	0	16	NA	-	-	-	-	-	-	-	-	-	-	-	-	-	-	-
South Africa	12	-	-	-	-	-	-	-	-	-	-	-	-	-	-	-	-	-	-	-	-	-	-	-	-	-	-
	-	1	2013	12	0	3	9	0	0	0	48	55	6	5	1	0	0	0	-	-	-	-	-	-	-	-	-
South Sudan	2	-	-	-	-	-	-	-	-	-	-	-	-	-	-	-	-	-	-	-	-	-	-	-	-	-	-
	-	1	2013	2	1	1	0	0	0	0	10	13	2	0	0	0	0	0	-	-	-	-	-	-	-	-	-
Swaziland	9	-	-	-	-	-	-	-	-	-	-	-	-	-	-	-	-	-	-	-	-	-	-	-	-	-	-
	-	1	2010	1	1	0	0	0	0	0	10	24	1	0	0	0	0	0	1	46	22	1	0	0	0	0	0
	-	2	2012	8	0	5	3	0	0	0	7	21	8	0	0	0	0	0	8	50	29	5	3	0	0	0	0
Tanzania	30	-	-	-	-	-	-	-	-	-	-	-	-	-	-	-	-	-	-	-	-	-	-	-	-	-	-
	-	1	2010	12	0	2	5	4	1	0	24	30	11	1	0	0	0	0	12	62	32	5	1	5	1	0	0
	-	2	2012	18	1	14	2	1	0	0	36	36	15	2	1	0	0	0	-	-	-	-	-	-	-	-	-
Uganda	76	-	-	-	-	-	-	-	-	-	-	-	-	-	-	-	-	-	-	-	-	-	-	-	-	-	-
	-	1	2011	21	3	5	10	3	0	0	52	34	17	1	2	1	0	0	21	66	32	7	2	5	3	3	1
	-	2	2013	55	2	6	47	0	0	0	126	35	49	2	3	0	1	0	-	-	-	-	-	-	-	-	-
Vietnam	12	-	-	-	-	-	-	-	-	-	-	-	-	-	-	-	-	-	-	-	-	-	-	-	-	-	-
	-	1	2012	12	0	6	6	0	0	0	24	44	10	2	0	0	0	0	12	78	34	1	2	2	6	1	0
Zambia	17	-	-	-	-	-	-	-	-	-	-	-	-	-	-	-	-	-	-	-	-	-	-	-	-	-	-
	-	1	2010	13	4	7	0	1	1	0	17	38	11	1	0	0	0	1	13	51	12	9	1	3	0	0	0
	-	2	2012	13	5	8	0	0	0	0	50	46	12	0	1	0	0	0	-	-	-	-	-	-	-	-	-
Zimbabwe	32	-	-	-	-	-	-	-	-	-	-	-	-	-	-	-	-	-	-	-	-	-	-	-	-	-	-
	-	1	2010	11	6	3	0	2	0	0	22	20	11	0	0	0	0	0	11	54	34	6	4	0	1	0	0
	-	2	2012	8	1	2	5	0	0	0	68	24	8	0	0	0	0	0	-	-	-	-	-	-	-	-	-
	-	3	2012	13	0	0	0	13	0	0	16	NA	-	-	-	-	-	-	-	-	-	-	-	-	-	-	-

**Total**	**617**	**63**	-	**654**	**118**	**176**	**250**	**63**	**32**	**15**	**1923**	-	**534**	**52**	**29**	**16**	**3**	**1**	322***	-	-	**93**	**69**	**63**	**53**	**37**	**7**
**Percent**	-	-	-	-	**18**	**27**	**38**	**10**	**5**	**2**	-	**37**	**84**	**8**	**5**	**3**	**0**	**0**	**49**	**65**	**29**	**29**	**21**	**20**	**16**	**11**	**2**
**Average**	-	-	-	**10.4**	-	-	-	-	-	-	**30.5**	-	-	-	-	-	-	-	-	-	-	-	-	-	-	-	-

-	-	-	-	**(Median *7,* range 1-55)**	-	-	-	-	-	-	**(Median 24, range 6-126)**	-	-	-	-	-	-	-	-	-	-	-	-	-	-	-	-

1 Caribbean Region: Bahamas, Jamaica, Barbados, Trinidad and Tobago (cohort #1) and St Vincent, St Kitts, Suriname, Grenada, Dominica, Antigua, St Lucia (cohort #2).

2 Central America Region: Costa Rica, El Salvador, Guatemala, Nicaragua, Panama.

3 US-DOD/NMRU: Belize, Columbia, Dominican Republic, El Salvador, Guatemala, Honduras, Nicaragua, Peru.

* Excluding repeat laboratories participating in multiple cohorts.

** Amongst cohorts with both baseline and exit audit results.

*** 302 Unique laboratories, plus 18 laboratories in Lesotho that have participated in multiple cohorts.

NA: Baseline scores not available.

**Total 47 Countries.**

As of December 2013, 65 SLMTA cohorts had been initiated, with one to 55 laboratories per cohort. Thirty were first cohorts within countries, including three regional cohorts which encompassed multiple countries, whilst the remaining 35 were subsequent cohorts in 23 countries as they expanded the programme (Table 1).

A total of 1923 people from 617 laboratories were trained. Thirty-seven of these laboratories (6%) were re-enrolled in a subsequent SLMTA cohort. Eighteen per cent of the 617 laboratories were at the national level, 27% at regional or provincial levels, 38% at district or primary levels, 10% belonged to non-governmental, faith-based or private organisations, 5% were military laboratories and 2% were blood banks. Nearly all (98%) of these laboratories provide HIV-related services, such as HIV diagnosis, treatment monitoring, opportunistic infection or tuberculosis testing, and blood bank testing. Of these laboratories, 302 (49%) completed the SLMTA programme and conducted an exit audit, whilst the remaining 315 (51%) were still going through the programme at the time of this analysis.

### Audit results

**FIGURE 2:**
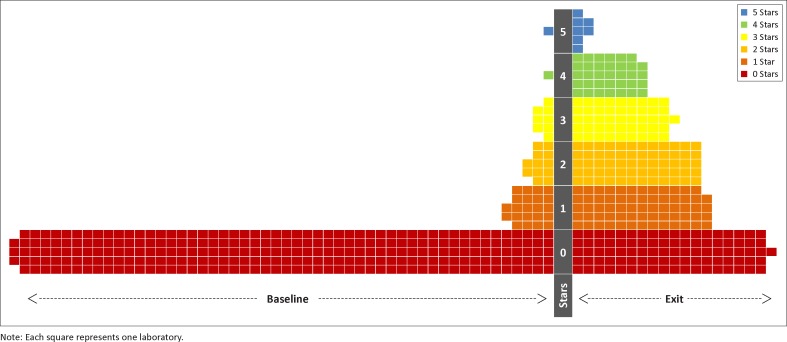
Star levels at baseline and exit amongst the 302 laboratories completing the Strengthening Laboratory Management Toward Accreditation programme, 2010–2013. Results based on the Stepwise Laboratory Quality Improvement Process Towards Accreditation checklist baseline and exit audit scores.

At the baseline audit, the mean score for all 617 SLMTA-enrolled laboratories was 39% (median 37%) and 84% received zero stars (i.e., score < 55%) on the SLIPTA five-star scale (Table 1). For the 302 laboratories that had completed the programme and conducted an exit audit, mean scores increased from 39% at baseline to 64% at exit (*p* < 0.001). Whilst 85% had received zero stars at baseline, only 30% remained at zero stars at exit (Figure 2).

The average improvement from baseline to exit audit was 25 percentage points over a mean programme duration of 16 months. Sixty-eight per cent of laboratories improved by at least one star, including 22% that increased by three or more stars. Although 32% of the laboratories did not increase their stars, 23% of these more than doubled their scores from baseline to exit. Improvements tended to be higher for laboratories with lower baseline scores. Those with a starting score below 25% had an average increase of 37 percentage points, whilst those with a starting score of 65% or higher had an average increase of nine percentage points (*r* = -0.49, *p* < 0.001) (Table 2).

**TABLE 2:** Improvement by baseline level based on the Stepwise Laboratory Quality Improvement Process Towards Accreditation checklist.

Baseline level	*n*	Mean baseline audit score (%)	Mean exit audit score (%)	Mean change (percentage points)
0% – 24%	59	19	56	37
25% – 34%	66	30	56	26
35% – 44%	73	39	62	23
45% – 54%	59	48	68	20
55% – 64%	23	59	79	20
65% +	22	75	84	9

**Total**	**302**	**39**	**64**	**25**

NGO, non-governmental organisation.

**FIGURE 3:**
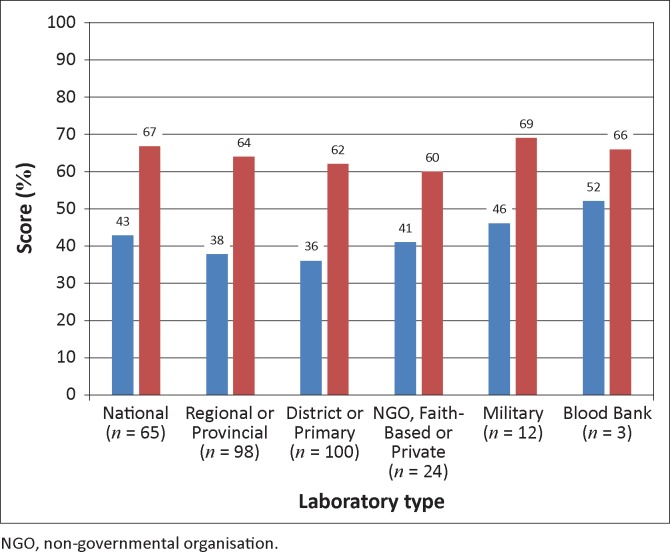
Baseline and exit scores by laboratory type for the 302 laboratories completing the Strengthening Laboratory Management Toward Accreditation programme 2010–2013 based on the Stepwise Laboratory Quality Improvement Process Towards Accreditation checklist.

Baseline and exit scores and improvements were similar between the different types of laboratories (*p* > 0.05) (Figure 3). Amongst laboratories that had completed the SLMTA programme, the 132 laboratories implementing SLMTA in the first year (2010) had the same mean improvement as the 170 laboratories implementing SLMTA in 2011 to 2013 (24 percentage points).

### Sustainability

**FIGURE 4:**
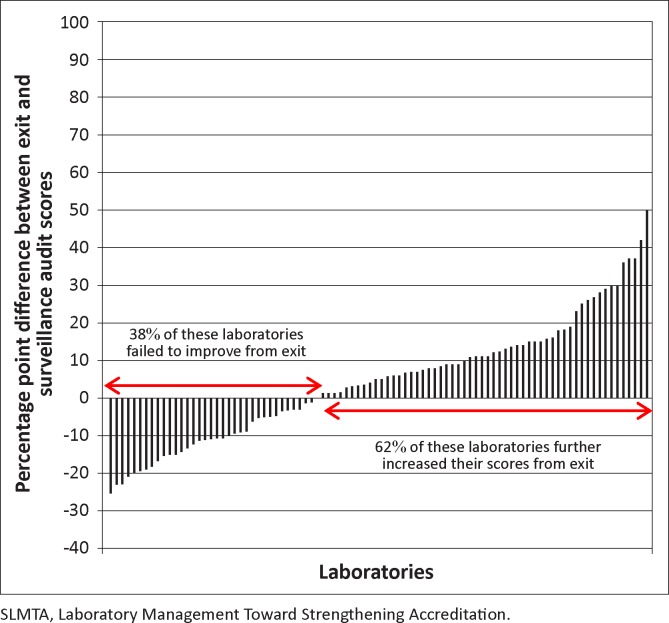
Post-SLMTA surveillance audits (*n* = 92 laboratories), based on the Stepwise Laboratory Quality Improvement Process Towards Accreditation checklist.

Ninety-two laboratories had conducted post-SLMTA surveillance audits, at a median of 11 months post-exit audit (range five to 28 months). Of these laboratories, 62% further increased their score, including 34% whose score increased by > 10 percentage points post-exit audit (Figure 4). Of the national-level laboratories that conducted post-SLMTA audits (*n* = 19), 79% improved their scores, whilst only 56% of district-level laboratories (*n* = 27) further increased their scores (*p* = 0.03). Overall, there was regression toward the mean, as laboratories that received lower scores at exit were more likely to improve their scores at the post-SLMTA audit (*r* = -0.48, *p* < 0.001).

As of September 2014, six SLMTA laboratories have been accredited to international standards (Table 3). These laboratories had baseline scores approximately equal to all SLMTA laboratories, with a median score of 38% (range 29% to 75%) as compared to a median of 37% for all SLMTA laboratories; one of the laboratories started at three SLIPTA stars, whilst the other five laboratories began at zero stars. At the exit audit, the six laboratories had a median score of 90%, with an increase of 52 percentage points, as compared to the international average of 64% and 25 percentage points. The median time from SLMTA initiation to accreditation was 31.5 months (range 17–54). In March 2013, the Kenya HIV National Reference Laboratory became the first SLMTA laboratory (and the first public medical laboratory in Kenya) to earn accreditation.^8^ The Hai Duong Preventive Medicine Center in Vietnam had the lowest baseline score of the six laboratories at 29%, but achieved the greatest improvement from baseline to exit (58 percentage points); it took the laboratory only 19 months from initiation of SLMTA to accreditation.

**TABLE 3:** Strengthening Laboratory Management Toward Accreditation laboratories that have achieved formal accreditation as of September 2014.

Laboratory name	Type of laboratory	Accrediting body	Date accredited	Date enrolled in SLMTA*	Time from SLMTA enrollment to accreditation	Baseline audit score (%)	Exit audit score (%)
Kenya HIV National Reference laboratory	National	Kenya Accreditation Service (KENAS) – ISO 15189	March 2013	April 2010	35 months	45	95
The Kenya Medical Research Institute (KEMRI)/CDC Tuberculosis Laboratory	National	The South African National Accreditation System (SANAS) – ISO 15189	July 2013	February 2012	17 months	75	95
Bahamas HIV National Reference Laboratory	National	College of American Pathologists (CAP)	September 2013	May 2011	28 months	38	63
Hai Duong Preventive Medicine Center, Vietnam	Regional/Provincial	Bureau of Accreditation, Vietnam – ISO 17025	December 2013	May 2012	19 months	29	87
The Cimas Harare Medical Laboratory, Zimbabwe	Private	The Southern African Development Community Accreditation Services (SADCAS) – ISO 15189	September 2014	March 2010	54 months	34	54
Bungoma District Hospital Laboratory, Kenya	District	KENAS – ISO 15189	September 2014	February 2011	43 months	38	92

SLMTA, Strengthening Laboratory Management Toward Accreditation; ISO, International Organization for Standardization; CDC, US Centers for Disease Control and Prevention; *, Date of first SLMTA workshop.

### SLMTA implications for laboratory testing

**FIGURE 5:**
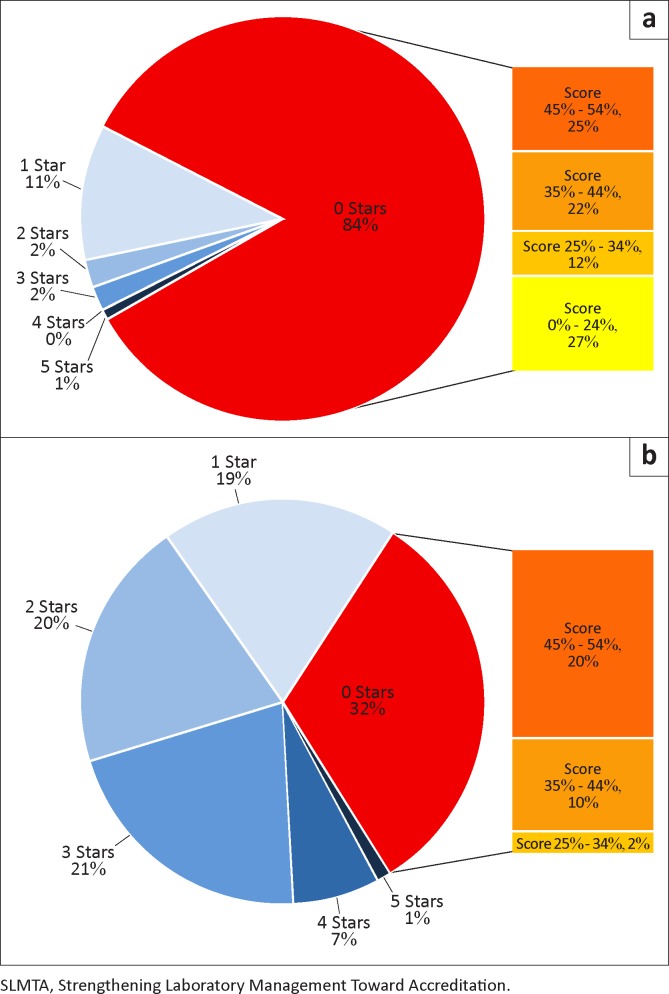
Estimated proportion of 43.5 million tests performed by star level of laboratories before (a) and after (b) SLMTA implementation, based on the Stepwise Laboratory Quality Improvement Process Towards Accreditation checklist scores for the 302 laboratories that completed the SLMTA programme. Before SLMTA: 16% of tests were done in laboratories with at least one star. After SLMTA 68% of tests were done in laboratories with at least one star.

In total, the 617 SLMTA laboratories conducted an estimated 111 million tests in 2012. Approximately 43.5 million of these tests were conducted by the 302 laboratories that had completed the SLMTA programme by the time of this analysis. Only 16% of these tests were conducted by laboratories with at least one quality star prior to SLMTA implementation (3% had ≥ 3 stars); after SLMTA training 68% of tests were performed by laboratories with at least one quality star (28% had ≥ 3 stars). This translates to approximately 23 million tests conducted by laboratories that previously had zero stars and now have one or more stars. Furthermore, within the group with zero stars prior to SLMTA implementation, more than one in three tests were performed by laboratories scoring below 35% on the SLIPTA checklist; after SLMTA completion this proportion decreased to one in 50 (Figure 5). Assuming similar quality improvements for the 315 currently-enrolled laboratories, we project the total number of tests conducted by laboratories with one or more stars that had previously been at zero stars to rise to 58 million by the end of 2015.

## Discussion

Since its launch in 2009, SLMTA has achieved wide global coverage and demonstrated measurable improvement using a standardised checklist, transforming the laboratory landscape in many resource-limited countries worldwide. The results have been remarkable, with audit scores increasing 25 percentage points, and two-thirds of the laboratories that completed the SLMTA programme improving by at least one quality star level. Twenty-three countries have implemented a second cohort of training and 23 million tests conducted by laboratories previously lacking any quality management system are now being conducted by laboratories with at least a basic level of quality in place. The SLMTA programme has the potential to make a substantial and sustainable impact on the quality of laboratory testing and, therefore, patient care.

As of August 2014, 102 laboratories from 15 countries in sub-Saharan Africa had received an official WHO AFRO SLIPTA audit by the African Society for Laboratory Medicine (ASLM) (Maruta T, 2014, personal communication, August 31); ninety-seven (95%) of these laboratories had gone through the SLMTA programme. It is evident that SLMTA has emerged as a vital strategy in accelerating laboratory preparedness for accreditation in the developing world and in facilitating the fulfillment of ASLM’s 2020 Vision, Goal 2: ‘by 2020, enroll 2500 laboratories in the WHO SLIPTA quality improvement programme and enable 250 laboratories to achieve accreditation by international standards’.^9^

To date, the SLMTA programme has been primarily funded by the US President’s Emergency Plan for AIDS Relief (PEPFAR) through CDC and implementation has been limited primarily to PEPFAR-supported countries. Although HIV-focused, these resources have been leveraged to build wide-spread capacity and strengthen the overall laboratory system.^10^ Slowly, partners are joining ranks to help spread the programme. Since 2011, the World Bank has funded five countries (Burundi, Kenya, Rwanda, Tanzania and Uganda) to implement SLMTA in 32 laboratories through its East Africa Public Health Laboratory Network Project. The Southern African Development Community (SADC) will soon partner with the Zimbabwe National Quality Assurance Program in spreading the programme to all 15 member states, some of which are not supported by PEPFAR.

This is the first study to examine the existing level of laboratory quality on a broad international scale. The mean baseline audit score of the 617 SLMTA-enrolled laboratories was 39%, well below the 55% required to attain even the lowest quality level on the SLIPTA scale, and only 16% of the laboratories met or surpassed the one-star level. These results paint a bleak picture of the current quality of laboratories in much of the developing world. Whilst the issue of the lack of quality amongst laboratories in developing countries has been acknowledged in numerous publications^4,11,12,13,14,15,16,17^ and a series of policy statements,^18,19,20,21^ the current study begins to quantify the problem and sheds some light on a potential solution.

The SLMTA curriculum was designed to be closely aligned to ISO 15189; however, it has proven to be suitable to support other standards as well. For example, a SLMTA-enrolled laboratory in Vietnam that tests environmental and food samples was accredited to ISO 17025. Two per cent of SLMTA sites are blood banks or blood-transfusion centres, which use different standards and have their own accreditation programmes. In Cameroon, inspired by the transformation achieved by its SLMTA-enrolled laboratory, a hospital adopted SLMTA in order to embark on its own quality improvement journey.^22^ The need for improved quality is so widespread that others have called for the expansion of SLMTA into a programme that helps guide quality improvement in entire hospital and public health systems.^23^

According to a May 2013 survey, only two government-owned medical laboratories in the 48 sub-Saharan African countries outside of South Africa were accredited.^11^ In 2013 to 2014, three more government-owned laboratories in the region (all located in Kenya) were accredited after SLMTA implementation (Table 3). In the same timeframe, two SLMTA laboratories outside of the sub-Saharan region have achieved accreditation, whilst several others are awaiting accreditation assessments. These are only the first of what we anticipate will be many SLMTA laboratories reaching accreditation as the programme matures, since preparing for accreditation is a multi-year process. However, it is not feasible, either economically or programmatically, for all laboratories to pursue accreditation to international standards. As countries develop laboratory strategic plans, they will need to assess the options and develop realistic goals. The Ministry of Health in Uganda, for example, has set a national target of three stars for general hospital laboratories and five stars for national and regional reference laboratories (Lali W, 2014, personal communication, August 31). Regardless of the ultimate goal, the SLMTA programme will provide the tools necessary to guide laboratories in the continuous quality improvement process designed to achieve better patient care.

It is outside the scope of this study to examine how factors such as cohort size, programme length, laboratory type, mentorship model (amount, quality and type) and additional training impact the performance of the programme. However, detailed data are being collected as part of the SLMTA programme, including audit scores for each of the 12 QSEs and laboratory indicators such as turnaround time, specimen rejection rates, equipment downtime, proficiency testing, customer satisfaction and cost. These data are a potential gold mine of information that could be harnessed to identify causative factors of success and to fine-tune the programme with evidence-based strategies for continued improvement. An electronic-tool that will facilitate collection, management, aggregation, analysis and reporting of SLMTA programmatic data on a global level is currently under development by CDC and its partners.

### Limitations of the study

These study results should be interpreted in light of several limitations. Firstly, half of the enrolled laboratories have not yet completed the programme and therefore have no exit audit data. As the programme matures, a higher proportion of the participating laboratories will have full data, reducing the risk of bias. Similarly, whilst we found that most laboratories that conducted surveillance audits continued to improve over a time-span of anywhere from five to 28 months after the SLMTA programme ended, the programme is too young to assess long-term sustainability. Another concern is the quality of the audits, especially in the initial years of the SLMTA programme before formal training and certification of auditors. Whilst the SLIPTA checklist was designed to help standardise audit scoring, some variability may remain; assessment of intra- and inter-auditor variability is needed.

This was an observational programmatic study. Many factors were not controlled, such as programme duration, size of cohorts, mentorship model and additional training provided to enrolled laboratories. Future studies that compare these factors, as well as results of SLMTA laboratories with non-SLMTA laboratories are needed in order to separate the impact of SLMTA implementation from natural improvement in laboratory quality over the duration of the programme. In addition, estimates of the number of laboratory tests were based on 353 (57%) laboratories that submitted information; missing data were imputed using average volume by laboratory type, but some uncertainty remains.

Ultimately, a thorough evaluation of the SLMTA programme will require assessment of programme impact on patient care and health outcomes. Systematic evaluation of key indicators is needed, as well as targeted evaluations of programme cost-benefit, the impact of quality improvement on testing error rates and the association of quality services with patient outcomes, so as to determine public and personal health implications.

## Conclusion

Few management and leadership development programmes have been implemented on such a large scale with results-oriented outcome measures. With data collected from 617 laboratories in 47 countries throughout Africa, the Caribbean, Latin America and Southeast Asia, SLMTA is truly a global effort. It has demonstrated its ability to transform the laboratory landscape in resource-limited countries worldwide. Evidence from this study suggests that the SLMTA programme has the potential to make a substantial and sustainable impact on the quality of laboratory testing and patient care.

## Appendix: Author information

**TABLE:** Group Authorship.

Country	Author #1 Name	Author #2 Name	Grant no.
Angola	Margarida Rodrigues	Filomena Gomes da Silva	AFENET 5U2GPS002728
Botswana	Kelebeletse Mokobela	Mpho Moatshe	GH000073
Burundi	Stanislas Nyandwi	Jean Marie Vianney Ngendakabaniga	East African Public Health Laboratory Networking Project
Cambodia	Chuop Sokheng	Uch Monipheap	PS000939
Cameroon	Judith D. Shang	Laura Takang Eno	3U2GPS002739-03S1
Caribbean	George Alemnji	Giselle Guevara	(AFENET)PS10-10119
Central America Region	Sandra Juarez	Ana Malla de Abreu	5U19GH00064
Cote d’Ivoire	Christiane Adje Toure	Ramatou Toure Hamidou	ASCP U2GPS001285-06
Dominican Rep	Ana Malla de Abreu	Rosa Hazim	FIND: U2GPS002746-03
D.R. Congo	Abiola Nadine	Kabwe Constantin	U2G PS001799 (APHL)
Ethiopia	Gonfa Ayana	Yenew Kebede	PS000825 (EHNRI)
			GH000204 (ICAP)
			HA06801 (ITECH)
			PS000858 (JHU)
			GH000303 (UCSD)
Ghana	Veronica Bekoe	Beatrice van der Puije	PS10-1070 U2GPS002762 5U2GPS002987
Haiti	Josiane Buteau	Mary Nagel	U2GPS001285-06
Kenya	Ernest Makokha	Jane Mwangi	ASCP: Grant # PS001285
			AGHPF: Grant # PS001865
			AFENET: Grant # PS002728
Lesotho	Mary Sekautu	Mathabo Lebina	CoAg# PS002076
Malawi	Elde Paladar	Henry Limula	3U2GPS001938-04
Mozambique	Eduardo Samogudo	Antonio Assane	ASCP- 5U2GPS001285-01
			INS – 1U2GGH000080-01
			APHL- U2G/PS001799-03
Namibia	Mary Mataranyika	Souleymane Sawadogo	MoHSS - CDC-RFA- PS10- 1095
Nigeria	Odafen Oke	Emmanuel Oni Idigbe	APIN: PS001058;
			IHNV: PS002929;
			FHI360/SIDHAS:
			AID-620-A-11-00002;
			DoD-NA
Rwanda	Jean Marie Vianney Ngendakabaniga	Alida Ngwije	U2GPS000642
Sierra Leone	Isatta Wurie	Fay Rhodes	APHL U2GPS001799
South Africa	Patience Dabula	Zawadi Chipeta	U2GPS001328-05
South Sudan	Lucy A. Mambo	Hakim Idris	U2GPS001799
Swaziland	Samson Haumba	Makhosanana Shabalala	3U2GPS001896
Tanzania	Dickson Majige	Abdul Mwanja	5U2GPS001788
Uganda	William Lali	Kamaranzi Bakunda	PS002724: CDC-RFA-PS10-102603
Vietnam	Kyle Bond	Cuong N. Duong	VAMS: PS001928
			FHI : PS001172
			ASCP: PS001285
			VAAC/LIFE-GAP: PS07-703
			APHL- U2G/PS001799-03
Zambia	Fales Zulu Mwamba	Davy Nsama	MOH CoAg Grant # U2GPS001792
			APHL CoAg Grant # U2GPS001799
			ASM CoAg Grant # U2GPS001947
Zimbabwe	Sibongile Zimuto	Phoebe Nzombe	3U2GPS003124

**Organisation**			

CDC-Atlanta	John Nkengasong	Luciana Kohatsu	-
Mayo Clinic	Barbara McKinney	-	-
American Society for Clinical Pathology (ASCP)	Anna Murphy	Mary (Kitty) Linde	-
African Society for Laboratory Medicine (ASLM)	Talkmore Maruta	Nqobile Ndlovu	-
Botswana Harvard Partnership, HIV Reference Laboratory	Sikhulile Moyo	-	-
East, Central and Southern African Health Community (ECSA-HC)	Martin Matu	-	-
Naval Medical Research Unit 6 (NAMRU-6)	Silvia Montano	Drake H. Tilley	Work Unit Number 60150
